# Poisoning by Organic Compounds of Arsenic

**Published:** 1910-07-23

**Authors:** 


### Toxicology.

### POISONING BY ORGANIC COMPOUNDS OF ARSENIC.

The toxicity of the organic compounds of arsenic of the atoxyl and soamin class being so very much less than is that of arsenic in the form of either of its liquors, there has arisen a tendency to think that atoxyl can be given almost with impunity. Small wonder is it, therefore, that cases are beginning to be recorded in which not merely toxic but actually fatal results have followed its administration. This does not indicate in the least that atoxyl and its congeners are bad drugs, or even dangerous in the ordinary sense. There are far more dangerous drugs in constant use, strychnine for example; but it is most important to be very careful in regard to all these potent drugs, and to remember that atoxyl, though relatively non-toxic, may actually be very toxic indeed. A paper by Dr. Heinrich Schlecht of Breslau contains details of a case in point. A young butcher, twenty-eight years of age, and hitherto perfectly healthy, developed a chancre, followed ten weeks later by typical secondary syphilis with rash and anal condylomata. Mercury was given at this stage by inunction, this method of administration being changed in three weeks' time for intramuscular injections of salicylate of mercury, with iodide of potassium by the mouth. At the end of another week, during which time a drachm and a quarter of iodide of potassium had been given each day, atoxyl injections were resorted to as well. During eight days the man received about 36 grains of atoxyl in four separate doses. On the ninth day serious symptoms set in, and the atoxyl was stopped. There was a sudden rise of temperature with vomiting, prostration, and obvious illness, followed a few hours later by coma, during which tonic and clonic spasms of the muscles more or less of an epileptiform nature supervened. The face and extremities became cyanosed; the respirations were very variable, being sometimes rapid and shallow, sometimes deep and slow, sometimes in abeyance altogether, as if death had actually ensued. The pulse was rapid and feeble. Albuminuria developed, the urine also containing renal tube casts. Death occurred during one of the epileptiform seizures; indeed, the picture drawn by Dr. Schlecht is highly suggestive of acute uræmia, as though the atoxyl had caused an acute parenchymatous nephritis.

A post-mortem examination was allowed, and the following changes were found: The kidneys were large, soft, and congested. The liver was fatty and it also exhibited areas of necrosis microscopically. The lungs were œdematous. The heart muscle presented marked degenerative changes histologically. The nervous system showed no distinctive lesions, but there was decided evidence of acute blood destruction both in the liver and in the spleen. All these changes indicate the action of a toxin either of microbial or of chemical origin. It might be urged that it was not the atoxyl itself that had acted as the poison, but rather some decomposition product of it; this may be so, but it nevertheless indicates one of the dangers of using atoxyl. It might also be urged that the patient's symptoms were due to an intercurrent microbial malady, or even to the virulence of the syphilis, and not to the atoxyl at all. This might be difficult to disprove, but the above case is by no means the only one in which similar symptoms have arisen after the administration of atoxyl. Koch observed over twenty cases of blindness after its use in 1,633 cases of sleeping sickness, the eye lesion being due either to retinal hæmorrhage or to optic atrophy. In different cases different symptoms predominate, but amongst those that have been traced to atoxyl may be mentioned albuminuria, hæmaturia, dysuria, actual nephritis, anorexia, vomiting, diarrhœa, jaundice, precordial pain, bradycardia, angina pectoris, vertigo, insomnia, pains in the limbs, alterations in cutaneous sensibility, and various skin rashes.

This list may seem so formidable that atoxyl might perhaps be regarded as altogether too dangerous to use. This is by no means necessarily the case, however. We would only urge that it should not be resorted to when simpler remedies might suffice, and that its dosage should be small to begin with until the way the patient bears it can be ascertained by observation.

